# Development and Validation of a Machine Learning Model to Aid Discharge Processes for Inpatient Surgical Care

**DOI:** 10.1001/jamanetworkopen.2019.17221

**Published:** 2019-12-11

**Authors:** Kyan C. Safavi, Taghi Khaniyev, Martin Copenhaver, Mark Seelen, Ana Cecilia Zenteno Langle, Jonathan Zanger, Bethany Daily, Retsef Levi, Peter Dunn

**Affiliations:** 1 Department of Anesthesia, Critical Care and Pain Medicine, Massachusetts General Hospital, Boston; 2 MIT Sloan School of Management, Massachusetts Institute of Technology, Cambridge; 3 Department of Perioperative Services, Massachusetts General Hospital, Boston

## Abstract

**Importance:**

Inpatient overcrowding is associated with delays in care, including the deferral of surgical care until beds are available to accommodate postoperative patients. Timely patient discharge is critical to address inpatient overcrowding and requires coordination among surgeons, nurses, case managers, and others. This is difficult to achieve without early identification and systemwide transparency of discharge candidates and their respective barriers to discharge.

**Objective:**

To validate the performance of a clinically interpretable feedforward neural network model that could improve the discharge process by predicting which patients would be discharged within 24 hours and their clinical and nonclinical barriers.

**Design, Setting, and Participants:**

This prognostic study included adult patients discharged from inpatient surgical care from May 1, 2016, to August 31, 2017, at a quaternary care teaching hospital. Model performance was assessed with standard cross-validation techniques. The model’s performance was compared with a baseline model using historical procedure median length of stay to predict discharges. In prospective cohort analysis, the feedforward neural network model was used to make predictions on general surgical care floors with 63 beds. If patients were not discharged when predicted, the causes of delay were recorded.

**Main Outcomes and Measures:**

The primary outcome was the out-of-sample area under the receiver operating characteristic curve of the model. Secondary outcomes included the causes of discharge delay and the number of avoidable bed-days.

**Results:**

The model was trained on 15 201 patients (median [interquartile range] age, 60 [46-70] years; 7623 [50.1%] men) discharged from inpatient surgical care. The estimated out-of-sample area under the receiver operating characteristic curve of the model was 0.840 (SD, 0.008; 95% CI, 0.839-0.844). Compared with the baseline model, the neural network model had higher sensitivity (52.5% vs 56.6%) and specificity (51.7% vs 82.6%). The neural network model identified 65 barriers to discharge. In the prospective study of 605 patients, causes of delays included clinical barriers (41 patients [30.1%]), variation in clinical practice (30 patients [22.1%]), and nonclinical reasons (65 patients [47.8%]). Summing patients who were not discharged owing to variation in clinical practice and nonclinical reasons, 128 bed-days, or 1.2 beds per day, were classified as avoidable.

**Conclusions and Relevance:**

This cohort study found that a neural network model could predict daily inpatient surgical care discharges and their barriers. The model identified systemic causes of discharge delays. Such models should be studied for their ability to increase the timeliness of discharges.

## Key Points

**Question:**

Can a neural network model predict which patients are likely to be discharged within 24 hours and their barriers to discharge?

**Findings:**

This prognostic study included 15 201 hospital discharges and found that the neural network model demonstrated an area under the receiver operating curve of 0.84 and strictly dominated a baseline model using median length of stay by surgical procedure type. The neural network model identified 65 barriers to discharge.

**Meaning:**

A neural network model predicted daily inpatient surgical care discharges and their barriers, which could be used by future clinicians to support efforts to increase the timeliness of patient discharge.

## Teaser

This prognostic study develops and validates the performance of a neural network machine learning model compared with a model based on median length of stay for predicting which patients are likely to be discharged within 24 hours from inpatient surgical care and their barriers to discharge.

## Introduction

As hospitals continue to experience high demand for their services, efficient capacity management is critical to fulfilling their mission to serve patients.^1,2,3,4^ Surgical teams’ ability to deliver timely operative care is reliant on the availability of postoperative floor beds. Furthermore, when inpatient beds are not available, patients needing transfer from outside hospitals to receive procedural care at large medical centers experience delays.

Timely patient discharge requires a coordinated effort among surgeons, nurses, case managers, physical therapists, and others. They must identify candidates for discharge early and the barriers that prevent the patient’s transition out of the hospital.^5,6,7,8^ Some hospitals request that the clinical teams identify and prioritize patients who will be discharged each day, although this task is manual, complex, and inconsistently performed.^9^ Without clearly identifying patients and their discharge barriers, successful coordination of discharge-related activities is difficult.

Few tools exist that are capable of automatically and accurately predicting which patients will be discharged and their barriers to discharge. Machine learning algorithms able to process substantial electronic clinical and operational data may offer an opportunity to create timely, systematic, and accurate predictions of which patients will be discharged.^10,11,12^ However, these algorithms must be combined with an interpretable clinical model to create an output that helps guide the clinical discharge processes. In doing so, such models can augment clinical teams by helping to focus their efforts on patients nearing discharge, explicitly identifying barriers to discharge that they can address, and creating transparency among multidisciplinary team members critical to the discharge process by communicating these elements automatically.

Using the clinical and operational data available in our hospital’s electronic medical record (EMR), we sought to develop a machine learning algorithm based on a clinical model that could successfully predict inpatient surgical care discharges. Our primary aim was to validate the performance of such a model in predicting inpatient surgical care discharges and barriers to discharge. Our secondary aim was to analyze the cause of cases in which the model predicted that the patient would be discharged but the patient remained in the hospital.

## Methods

The Partners Healthcare Human Research Committee reviewed and approved the study and access to EMR data for the purposes of this study. The need for oral or written consent from study participants was waived by the committee because data were deidentified and stored in secure, encrypted computers.

### Study Setting and Population

The study was conducted at a 1034-bed quaternary care teaching hospital where more than 36 000 surgical procedures are performed each year. During the study period, there was a mean of 151.8 patients discharged per day from the hospital, 31.2 of whom were patients who underwent inpatient surgical care.

The model was trained on all adult inpatients (except patients undergoing cardiac surgical care) aged 18 years or older who underwent a surgical procedure in the operating room, were cared for by a surgical team on a general surgical care inpatient floor, and were discharged between May 1, 2016, to August 31, 2017. The setting included 13 distinct inpatient surgical or medical-surgical units with a total of 360 beds. At the time of this study, a separate and distinct surgical team was responsible for patient flow among patients undergoing cardiac surgical care. Therefore, patients undergoing cardiac surgical care were not considered in-scope for this study. To test the performance of the model against a baseline model, we used a patient population with identical inclusion and exclusion criteria who were discharged from September 1, 2017, to December 31, 2017. In the prospective implementation of the model, we performed an observational cohort analysis on all postoperative inpatients admitted and discharged from 2 inpatient general surgery floors, including a total of 63 beds, during 104 days from January 8, 2018, to April 22, 2018.

### Data Sources

We used clinical and administrative data from an institutional database linked to patients’ EMRs. The data for the daily prediction incorporated all data until 23:59 on the day prior to prediction. This time was used because the clinical and administrative database was updated every 24 hours, starting at 23:59. We included demographic, environmental, administrative, and clinical data in the model (Table 1). Most data elements were structured, including either numerical values (eg, laboratory results) or multiple choice of predetermined text phrases (eg, diet descriptions within diet orders). Other data elements were in free-text form in case manager notes and required text processing to transform them into data elements usable by the model. We used a method of key word search in which we searched for the existence of a predefined list of words in the note such as, “home today,” “discharge today,” “is medically ready,” and “referral placed.”

**Table 1.** Overview of the Machine Learning Model Data Inputs

Clinical Data Category	Data Item
Patient demographic information	Age
	Sex
Surgery information	Surgical procedure type
	Surgical urgency
Clinician orders	Laboratory
	Radiologic
	Dietary
	Physician consultation
Clinical test results	Laboratory
	Radiologic
Bedside assessments	Vital signs
	Fluid intake
	Fluid output
	Cognitive assessments
Clinical recommendations	Physical therapist
	Case manager
	Speech language pathologist
Medication administration	Type of medication
	Dose administered
	Route of administration
Catheter information	Type of catheter
	Date and time of placement
	Date and time of removal
Care team notes	Physical therapy
	Case management

### Model Development

#### Clinical Milestones of Recovery and Barriers to Discharge

Clinical data were included in the model based on whether they represented a clinical milestone of recovery or a clinical or administrative barrier to discharge. A *clinical milestone of recovery* was defined as an event that marked the patient’s progress toward recovery and discharge. A *barrier to discharge* was defined as an event that may have postponed the patient’s discharge. Clinical milestones and barriers to discharge were identified by a multidisciplinary team that included physicians, nurses, case managers, physical therapists, and nutritionists. Milestones and barriers were recorded by a member of the study team and were ultimately included in the model based on whether data related to these events were available in the database linked to the patient’s EMR.

Clinical milestones could be specific to a type of surgical procedure or generalizable across multiple types of surgical procedures. Milestones were tracked by the model for changes in value that could be clinically interpreted as a milestone having been achieved. Milestones were defined in the following categories: transition from intravenous to oral medications; laboratory results within reference ranges; vital signs within reference ranges; completion of consult assessments; completion of imaging studies; removal of surgical drains, catheters, and wound devices; resolution of barriers to postacute care transitions; improvement in functional or mobility challenges; and nursing bedside assessments within reference ranges (eg, no cognitive abnormalities).

Potential barriers to discharge were defined in the following categories: medication administration; laboratory results outside of reference ranges; vital signs outside of reference ranges; awaiting specialist consult assessments; new scheduled surgical procedures; presence of surgical drains, catheters, and wound devices; postacute care transition barriers; functional or mobility challenges; and nursing bedside assessments outside of reference ranges (eg, cognitive abnormalities). Barriers to discharge were tracked by the model for changes in value that could be clinically interpreted as resolution.

#### Prediction Algorithm, Model Selection, and Barrier Identification

We elected to use a multilayer perceptron feedforward neural network model that predicts a continuous score between 0 and 1 associated with the likelihood of a patient to be discharged within 24 hours. The decision to use a neural network model was motivated by our assumption that the final decision to discharge a patient is associated with underlying clinical and nonclinical variables that interact in a complex, highly nonlinear fashion.^13^ Further, the neural network model supported online learning, so that when new data became available, they could be used to update the best prediction for future data in an adaptive manner. This allowed us to continue improving the prediction accuracy in an efficient manner.

The neural network model was trained on 15 201 patients who underwent inpatient surgical care and 75 582 predictions from May 1, 2016, to August 31, 2017 (eFigure 1 in the Supplement). The final model parameters were chosen using cross-validation over various network architectures (number of hidden layers and number of nodes in those layers) and regularization hyperparameters. The metric used for selection during cross-validation was the mean validation set area under the receiver operating characteristic curve (AUC). The final selected model was then trained using these parameters and the data for the entire cohort. eFigure 2 in the Supplement presents the patient populations and dates from which the training and validation sets were derived.

A set of candidate barriers to discharge was identified by manually selecting from the model’s variables those that pertain to a clinical or operational indicator related to discharge (eg, “Not taking a regular oral diet,” “Home visiting nurse services not available”). A candidate barrier was determined to be an actual barrier to discharge by systematically toggling the value of the variable and recalculating the prediction score by the model. If the new score was higher than the initial score, the candidate barrier was marked as an actual barrier for the patient.

### Statistical Analysis

#### Model Performance and Comparisons

We computed an estimated out-of-sample AUC by conducting 10 random experiments. In each, we randomly distributed the cohort into training (80%) and validation (20%) sets.^14^ We measured the testing accuracy of the model using the fixed parameters and randomly sampled training data.

To simplify the model output for clinical end-users into a binary classification, we determined an optimal threshold value above which we designated that a patient was predicted to be discharged and below which a patient was predicted not to be discharged. We defined the optimum threshold as that which would equally balance model sensitivity and positive predictive value (PPV). We calculated the threshold using experiments with the random validation samples in which we continuously adjusted the threshold score from 0 to 1 and identified the threshold at which the sensitivity and PPV were equal. Finally, we rounded the threshold to the nearest tenth digit. Using the binary output, we calculated the sensitivity, specificity, PPV, and negative predictive value (NPV).

We compared the neural network model relative to a baseline model that captures a simple discharge planning approach aligned with current practice at our institution. The baseline model used the historical median length of stay (LOS) of a given surgical procedure type to predict when the patient would be discharged. We calculated the sensitivity, specificity, PPV, and NPV of the median LOS model.

#### Prospective Observational Analysis of Discharge Delays

eFigure 2 in the Supplement presents the patient population and dates from which the prospective analysis of the model was derived. During the prospective analysis, discharge predictions were performed each day for all postoperative patients on 2 general surgical care floors that included 63 inpatient beds. Each day, candidates for discharge from the previous day who did not leave the hospital within 24 hours were discussed during a multidisciplinary team meeting. Multidisciplinary meetings were attended by representatives from surgery, nursing, physical therapy, case management, and social work. A member of the study team recorded the reasons that the team provided for why a patient was not discharged. In addition, a member of the study team (K.C.S.) performed an EMR review of each patient to confirm the stated reasons. Structured and unstructured data were reviewed. Unstructured data included physician and nursing progress notes written on the day of predicted discharge and on each subsequent day that the patient remained in the hospital.

Reasons for nondischarge were categorized into 3 areas (eTable 1 in the Supplement): (1) clinical barrier, (2) variation in clinical practice, or (3) no clinical reason. The no clinical reason group was subcategorized into (a) no reason identified, (b) delayed follow-up on patient progress by the care team, (c) care transition issues to home or facility, or (d) patient or family request to remain in the hospital. The number of estimated avoidable bed-days was calculated by summing the bed-days associated with the variation in clinical practice and no clinical reason categories.

For patients who were not discharged owing to variation in clinical practice or no reason identified, we determined whether there was a change in the patient’s treatment during their remaining hospitalization using EMR review. A *change in treatment* was defined as a change in medications, new procedures performed, new imaging studies performed or analyzed, or catheters placed or removed.

## Results

### Model Performance and Comparisons

**Figure 1.**
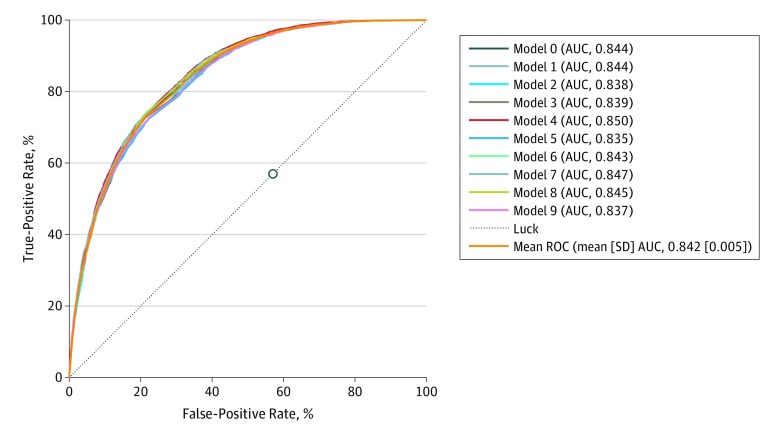
Receiver Operating Characteristic (ROC) Curve for Each Out-of-Sample Experiment in the Neural Network Model The circle represents the performance of the median length of stay (LOS) model. AUC indicates area under the ROC.

Patient characteristics for the cohort used to train and validate the feedforward neural network model are presented in Table 2. The training set included 15 201 patients (median [interquartile range] age, 60 [46-70] years; 7623 [50.1%] men), and the validation set included 3843 patients (median [interquartile range] age, 62 [49-72] years; 1882 [49.0%] men). Figure 1 presents the out-of-sample receiver operating characteristic curves and their AUC for each of the 10 experiments using the model. The mean AUC achieved by the neural network model was 0.840 (SD, 0.008; 95% CI, 0.839-0.844) (Figure 1).

**Table 2.** Machine Learning Model Training and Validation Cohort Characteristics

Characteristic	Patients, No. (%)	
	Training	Validation
Age, median (IQR), y	60 (46-70)	62 (49-72)
Sex		
Men	7623 (50.1)	1882 (49)
Women	7578 (49.9)	1961 (51)
Length of stay, median (IQR), d	3 (2-5)	3 (1-5)
Treating specialty		
Orthopedic surgery	4912 (32.3)	1234 (32.1)
Neurosurgery	2294 (15.1)	594 (15.4)
General surgery	1861 (12.2)	483 (12.5)
Urology	1189 (7.8)	310 (8.0)
Thoracic surgery	1014 (6.7)	259 (6.7)
Vascular surgery	969 (6.4)	213 (5.5)
Emergency general surgery	885 (5.8)	225 (5.8)
Surgical oncology	765 (5.0)	211 (5.5)
Plastic surgery	495 (3.3)	108 (2.8)
Transplant surgery	379 (2.5)	109 (2.8)
Oral maxillofacial surgery	221 (1.5)	55 (1.4)
Interventional radiology	130 (0.9)	23 (0.5)
Pediatric surgery	40 (0.3)	12 (0.3)
Other	47 (0.3)	7 (0.1)
Outcomes observed		
Not discharged	60 381 (79.9)	14 732 (79.4)
Discharged	15 201 (20.1)	3843 (20.6)
Most frequently observed barriers		
Case management assessed the patient to be high risk	44 826 (59.3)	11 672 (62.8)
Patient needs assistance to ambulate	39 002 (51.6)	8139 (43.8)
Patient requires services on discharge	35 031 (46.3)	9877 (53.1)
Imaging study not completed	26 091 (34.5)	7387 (39.7)
Patient not taking a regular oral diet	23 341 (30.8)	6056 (32.6)
Urinary catheter in place	20 003 (26.4)	5168 (27.8)
Systolic blood pressure >180 mm Hg within the past 24 h	17 418 (23.0)	4713 (25.3)
Patient currently needing oxygen supplementation	15 491 (20.4)	3724 (20.0)
Respiratory rate >30 breaths/min within the past 24 h	14 159 (18.7)	3615 (19.4)
Intravenous antiemetic administered within the past 24 h	9073 (12.0)	2340 (12.6)

Abbreviation: IQR, interquartile range.

The top 20 barriers in terms of relative weight on the model’s predictions are listed in Table 3. A complete list of the barriers and their weights are presented in eTable 2 in the Supplement.

**Table 3.** Top 20 Barriers by Relative Weight of Effect on the Machine Learning Model

Barrier	Weight	Patients, %
Patient not taking a regular oral diet	0.118	84.0
Home visiting nurse services not available	0.117	0.2
Patient lacks social supports	0.102	0.2
Occupational therapy recommends disposition to inpatient facility	0.040	15.4
Negative pressure wound vacuum in place	0.032	7.3
Fever occurring within the past 24 h	0.032	16.5
Epidural catheter in place	0.031	26.6
Biliary drain in place	0.03	4.6
Patient currently receiving IV antibiotics	0.025	1.7
Chest tube in place	0.021	3.6
Patient received a blood transfusion within the past 24 h	0.021	0.7
Patient currently receiving IV heparin infusion	0.020	1.7
Patient needs assistance to ambulate	0.018	83.6
Penrose drain in place	0.017	2.3
Urinary catheter in place	0.015	61.7
Suction drain in place	0.015	26.3
Impaired level of consciousness	0.015	22.5
Patient lacks financial resources	0.015	0.2

Abbreviation: IV, intravenous.

To measure model performance in terms of sensitivity, specificity, PPV, and NPV, the neural network model was tested on 3328 patients. The neural network had a sensitivity of 56.6%, a specificity of 82.6%, a PPV of 51.7%, and an NPV of 85.2%. Overall, 838 patients (27.8%) were discharged later than the neural network model predicted, including 461 patients (14.0%) discharged within 24 hours after predicted, 196 patients (5.9%) discharged between 24 to 48 hours after predicted, and 178 patients (5.3%) discharged more than 48 hours after predicted.

Figure 1 demonstrates a comparison between the neural network model’s performance and the median LOS model. The neural network model strictly dominates the median LOS model given that there is at least 1 point on the curve of the neural network model which is at least as good as the median LOS model in terms of true positive rate and strictly better than median LOS in terms of false positive rate, and vice versa. The median LOS model performance was worse than the neural network model in terms of sensitivity (52.5% vs 56.6%), specificity (51.7% vs 82.6%), PPV (25.6% vs 51.7%), and NPV (77.5% vs 85.2%). Overall, 1123 patients (33.7%) were discharged later than the median LOS model predicted, including 413 patients (12.4%) discharged within 24 hours after predicted, 217 patients (6.5%) discharged between 24 to 48 hours after predicted, and 493 patients (14.8%) discharged more than 48 hours after predicted.

### Prospective Observational Analysis of Discharge Delays

**Figure 2.**
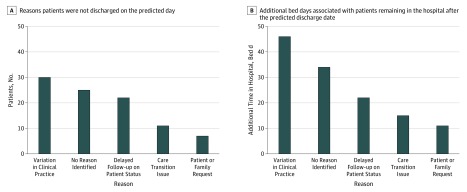
Causes of Patients Being Discharged Later Than Predicted

The prospective observational analysis was performed on 605 patients. Overall, 136 patients were discharged later than the model predicted. Figure 2 presents the reasons why patients were not discharged on the day they were predicted to by the model and the associated number of bed-days they remained in the hospital after this date, including 41 patients (30.1%) with clinical barriers, 30 patients (22.1%) with variations in clinical practice, and 65 patients (47.8%) with no clinical reason. Among patients with no clinical reason for delayed discharge, 25 patients (38.5%) had no reason identified, 22 patients (33.8%) had delayed follow-up on patient progress by the care team, 11 patients (16.9%) had care transition issues to home or facility, and 7 patients (10.8%) had a patient or family request to remain in the hospital.

Among 55 patients who remained in the hospital owing to variation in clinical practice or no clinical reason, there was no change in the clinical treatment of the patient from the predicted day of discharge to actual day of discharge for 47 patients (85.5%). Summing patients who were not discharged owing to clinical variation in practice and no clinical reason, the total estimated avoidable bed-days during the pilot study was 128 bed-days, or 1.2 beds per day.

## Discussion

This cohort study examined the performance of a feedforward neural network model to predict which patients who had undergone inpatient surgical care would be discharged 24 hours in advance and their respective clinical and nonclinical barriers. We found that the model demonstrated strong discrimination, with an AUC of 0.84, and strictly dominated a comparison model that used historical median LOS by surgical procedure type to predict discharges. In a prospective analysis of patients who were discharged later than the date the model predicted, we found that in most cases, patients remained in the hospital owing to nonclinical reasons or variation in clinical practice. These results were used to estimate potentially avoidable bed-days.

To our knowledge, this is the first study to demonstrate a model that can predict daily discharges at the individual patient level and automatically provide feedback on discharge barriers. Based on our findings, we believe that this model could aid clinicians in overcoming several challenges to timely discharges. First, it provided transparency into which patients were candidates for discharge within the next 24 hours, which can help clinical teams focus their efforts on appropriate patients each day. Second, it identified actionable clinical and nonclinical barriers. These functions can aid the coordination of discharge activities across the multidisciplinary team required to transition the patient out of the hospital. We intended for the model to facilitate targeted discussions regarding discharge and create alignment among the multidisciplinary clinical team tasked with discharging the patient. Importantly, the model was developed for the purpose of improving the discharge process rather than replacing the decisions of the clinical team. The model’s output is intended to interact with the knowledge of clinical team members not captured in the tool and, in combination, augment the discharge process.

Previous models have included variables that, while important to understanding the context of the patient regarding discharge, most often represented nonactionable or nonmodifiable factors, such as age, chief concern, day of the week, insurance status, and race/ethnicity.^15,16^ During this model’s development, we gathered the input of physicians, nurses, case managers, physical therapists, nutritionists, and others responsible for the care of the patient. These discussions led to the generation of additional features in the model and provided critical guidance as to which barriers were most actionable by the team and helpful to display.

In addition, the model identified patients who were not discharged when predicted, and we analyzed the causes of these nondischarges. This demonstrated the use of the model as a tool that could inform hospital process improvement and system redesign efforts regarding discharge. Typically, these efforts are challenged by the lack of a designated expected day of discharge. Thus, there is no mechanism to identify scenarios in which a patient was discharged later than expected. The current practice at our hospital is to ask nurses, case managers, or physicians to input expected days of discharge into the EMR; however, this is a manual, labor-intensive process and can vary across clinicians.

We found that the model could be used to focus the review and analysis of discharge delays on a subset of patients who were not discharged on the day predicted by the model. We found that these patients remained in the hospital for reasons that were likely addressable by the clinical teams. Most of these patients did not have substantially different or new medical treatment provided to them. Future research is needed to study the use of this tool by clinical teams in daily practice to determine whether this tool could help clinical teams decrease avoidable bed-days.

One challenge with this model is determining the threshold above which the patient is predicted to be discharged. We selected a threshold that balanced true-positives and false-positives. We found that the model had high specificity, although its sensitivity was relatively lower. Depending on what the clinical team is attempting to accomplish, the appropriate threshold may vary. For example, clinician users may want to choose a lower threshold that is more sensitive to ensure that they capture as many discharges as possible while tolerating some patients who may not yet be ready for discharge. Others in the hospital, such as bed managers, may prefer an approach that is highly specific as they may only want to know about beds that are certain to become available as they look to accommodate incoming patient demand.

Our findings add to the limited and emerging body of literature on machine learning applications designed to improve clinical operations. Previous studies have examined whether neural networks could be used to predict LOS. These models have typically been developed to predict LOS prior to admission to aid capacity planning by bed managers.^14,15,16,17^ By predicting LOS at a time remote from discharge, these models have typically not identified barriers to discharge or actions that the team could take in the moment to ensure timely discharge. In addition, to our knowledge, previous models have neither predicted discharges at a patient-level nor provided information regarding barriers to discharge that could guide clinical teams. Furthermore, some models that have predicted daily discharges have done so across all patients in the hospital, which is potentially useful at the hospital level but not actionable for clinical teams caring for the individual patient.^18,19^

### Limitations

Our study has some limitations. The model was trained and validated on patients within a single institution; thus, its generalizability to other hospitals is uncertain. We included demographic and clinical variables that are common to patients regardless of geographic location or health system; however, it is plausible that the model would behave differently in hospitals outside of our institution.

In addition, we validated the model’s performance by comparing it with a model that used median LOS of the patient’s type of surgical procedure to predict the day of discharge. We chose to use this model as a comparison because we believed it to be a reasonable approximation of how clinicians estimate the discharge date in their typical practice. This is particularly true of surgical procedures with standard pathways of recovery. For example, a patient who receives a laparoscopic sleeve gastrectomy in our institution is generally known to have an LOS of 1 day, and teams expect to discharge patients on this day. However, for patients who do not have standard pathways of recovery, historical median LOS may not be a good approximation of the expected discharge date.

The inputs for the model were limited to those discoverable on the EMR. If the data were not included in the EMR, such as verbal discussions about a patient’s status with regard to discharge, they could not be captured by the model. As a result, the model may underestimate or overestimate the likelihood of discharge and underreport important barriers to discharge.

## Conclusions

This study found that a clinically interpretable neural network model could predict daily inpatient surgical care discharges and their barriers. We used the model to identify causes of discharge delay. Such a model could potentially be used in the future to guide process improvement and increase the timeliness of discharges through daily prioritization of patients and barriers identification. These functions could aid hospitals in addressing capacity challenges and thereby reduce delays in patient care.

## Notes

Supplement. **eTable 1.** Categorized Reasons for Nondischarge and Definitions **eTable 2.** List of Barriers Detected by the Machine Learning Model and Their Relative Weight **eFigure 1.** Schema of Patients Included in the Training Set of the Neural Network Model **eFigure 2.** Patient Populations and Cohorts With Corresponding Dates
