# Supplementary material for: Development and Validation of a Machine Learning Model to Aid Discharge Processes for Inpatient Surgical Care
Source: JAMA Netw Open. 2019 Dec 11;2(12):e1917221. doi: 10.1001/jamanetworkopen.2019.17221 (PMC6991195; doi:10.1001/jamanetworkopen.2019.17221)
Supplement: Supplement. — eTable 1. Categorized Reasons for Nondischarge and Definitions eTable 2. List of Barriers Detected by the Machine Learning Model and Their Relative Weight eFigure 1. Schema of Patients Included in the Training Set of the Neural Network Model eFigure 2. Patient Populations and Cohorts With Corresponding Dates [file jamanetwopen-2-e1917221-s001.pdf]

Supplementary Online Content

Safavi KC, Khaniyev T, Copenhaver M, et al. Development and validation of a machine learning model to aid discharge processes for inpatient surgical care. *JAMA Netw Open*. 2019;2(12):e1917221. doi:10.1001/jamanetworkopen.2019.17221

eTable 1. Categorized Reasons for Nondischarge and Definitions

eTable 2. List of Barriers Detected by the Machine Learning Model and Their Relative Weight

eFigure 1. Schema of Patients Included in the Training Set of the Neural Network Model

eFigure 2. Patient Populations and Cohorts With Corresponding Dates

This supplementary material has been provided by the authors to give readers additional information about their work.

© 2019 Safavi KC et al. *JAMA Network Open.*

eTable 1. Categorized Reasons for Nondischarge and Definitions

**Reason**	**Definition**
Clinical barrier	a new clinical development occurring on the day of prediction that led to the team determining that the patient was not clinically ready for discharged
Variation in clinical practice	a clinical determination made by the team that the patient should not be discharged when such decision was determined to differ depending on the attending surgeon responsible for the patient
No clinical reason	no clinical reason identified
No reason identified	no clinical or non-clinical reason identified
Delayed follow-up on patient progress	when the clinical team had identified a singular barrier to discharge that was surmounted by the patient from 07:00 to 17:00 but the patient was not discharged
Care transition issue to home or facility	factors related to the home or facility's ability to receive the patient
Patient/family request to remain in the hospital	patient/family requested to be discharged on later date

© 2019 Safavi KC et al. *JAMA Network Open.*

eTable 2. List of Barriers Detected by the Machine Learning Model and Their Relative Weight

Barrier	Weight	Patients
Not taking a regular oral diet	0.118	84.0%
Home visiting nurse services not available	0.117	0.2%
Lacks social supports	0.102	0.2%
Occupational therapy recommends disposition to inpatient facility	0.04	15.4%
Negative pressure wound vacuum in place	0.032	7.3%
Fever occurring within the past 24 hours	0.032	16.5%
Epidural catheter in place	0.031	26.6%
Biliary drain in place	0.03	4.6%
Currently ordered for IV antibiotics	0.025	1.7%
Chest tube in place	0.021	3.6%
Received a blood transfusion within the past 24 hours	0.021	0.7%
Currently ordered for an IV heparin infusion	0.02	1.7%
Needs assistance to ambulate	0.018	83.6%
Penrose drain in place	0.017	2.3%
Urinary catheter in place	0.015	61.7%
Suction drain in place	0.015	26.3%
Impaired level of consciousness	0.015	22.5%
Lacks financial resources	0.015	0.2%
Patient/family having difficulty coping with illness	0.014	0.3%
Last WBC >12,000 cells/mL^3	0.013	31.6%
Needs assistance to transfer from surface to surface	0.01	17.7%
Currently needing oxygen supplementation	0.01	46.0%
Last blood glucose level >250 mmol/L	0.01	8.4%
Oxygen saturation <88% within the past 24 hours	0.01	4.3%
Needs assistance to stand	0.009	33.4%
Hemoglobin level <7.0 g/dL within the past 24 hours	0.009	14.4%
Uses a device to go from sitting to standing	0.008	28.6%
Not cognitively oriented	0.008	8.9%
Not active out of bed	0.007	36.4%
Nasogastric tube in place	0.007	23.3%
Potassium level >5.0 mEq/L within the past 24 hours	0.007	8.9%
Psychiatric/substance abuse history	0.007	2.3%
Needs assistance to sit	0.006	29.9%
Imaging study not completed	0.006	53.7%
Respiratory rate >30 breaths/min within the past 24 hours	0.005	38.0%
IV narcotic administered within the past 24 hours	0.005	33.7%
IV antiemetic administered within the past 24 hours	0.005	38.8%
IV antipsychotic administered within the past 24 hours	0.005	6.9%

© 2019 Safavi KC et al. *JAMA Network Open.*

Non-sinus rhythm recorded within the past 24 hours	0.005	3.0%
Limited home social support	0.005	2.0%
Patient/family prefer different facility	0.004	8.4%
Serum sodium >150 mEq/L within the past 24 hours	0.004	9.1%
Outpatient services not available	0.004	0.2%
Case management assessed the patient to be high risk	0.003	74.0%
Requires services on discharge	0.003	61.5%
Speech is unclear	0.003	6.6%
Requires soft restraints	0.003	5.8%
Systolic blood pressure >180 mmHg within the past 24 hours	0.002	52.2%
Abnormal creatinine within the past 24 hours	0.002	23.1%
Abnormal swallow study pharyngeal phase	0.002	5.0%
Requires long-term acute care facility	0.002	2.8%
IV diuretic administered within the past 24 hours	0.002	10.6%
Suprapubic catheter in place	0.002	0.2%
Physical therapy recommends facility	0.001	23.6%
Open surgical wound	0.001	3.0%
Guardianship issues	0.001	0.3%
Recommended for swallow evaluation	0	4.6%
Abnormal swallow evaluation response	0	4.6%
Abnormal swallow evaluation oral phase	0	4.5%
Abnormal swallow evaluation oral medication tolerance	0	4.6%
Abnormal cognition	0	4.3%
Insurance issues/uninsured	0	2.5%
Troponin elevated within the past 24 hours	0	2.0%
External fecal management system device in place	0	0.2%
IV, intravenous; WBC, white blood cell count; mL, milliliter; mmol, millimoles; L, liter; g, grams; dL, deciliters; mmHg, millimeters mercury		

© 2019 Safavi KC et al. *JAMA Network Open.*

eFigure 1. Schema of Patients Included in the Training Set of the Neural Network Model

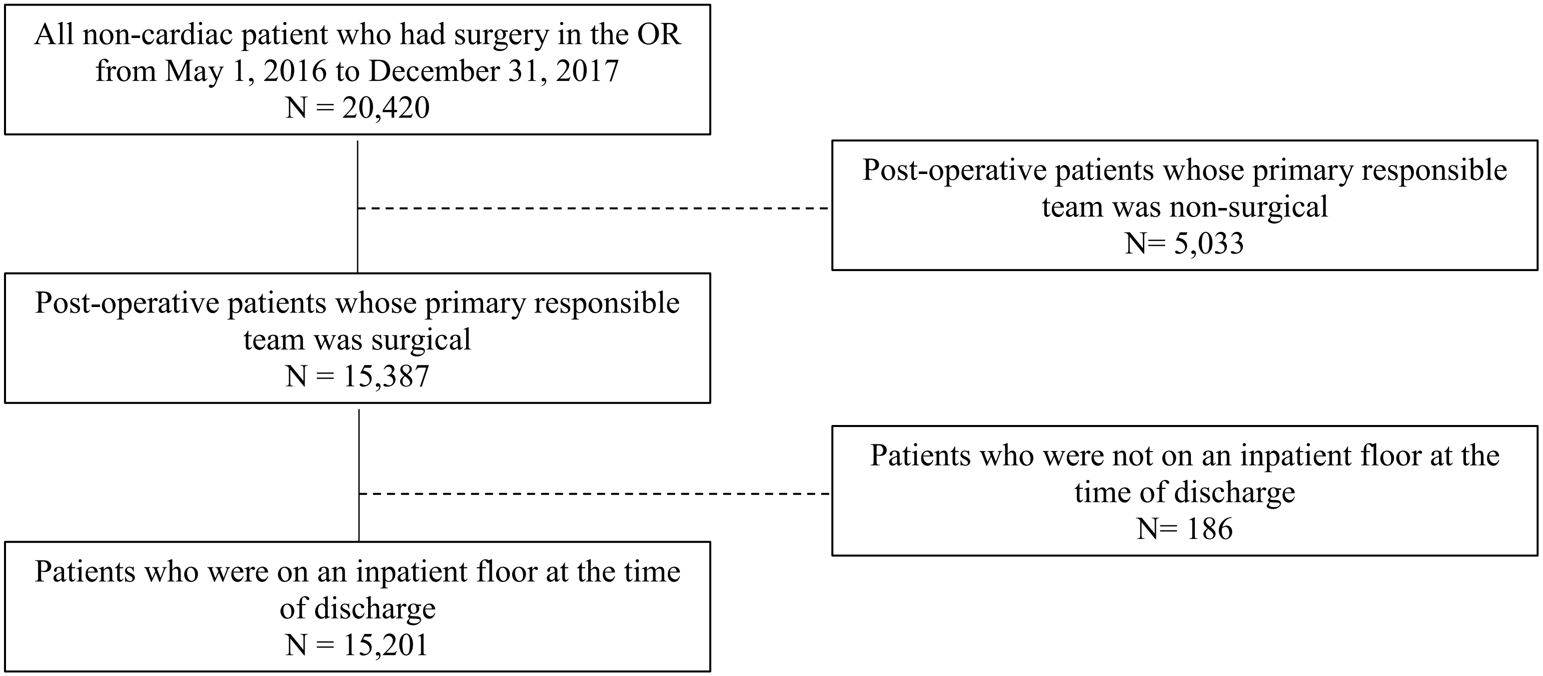

© 2019 Safavi KC et al. *JAMA Network Open.*

eFigure 2. Patient Populations and Cohorts With Corresponding Dates

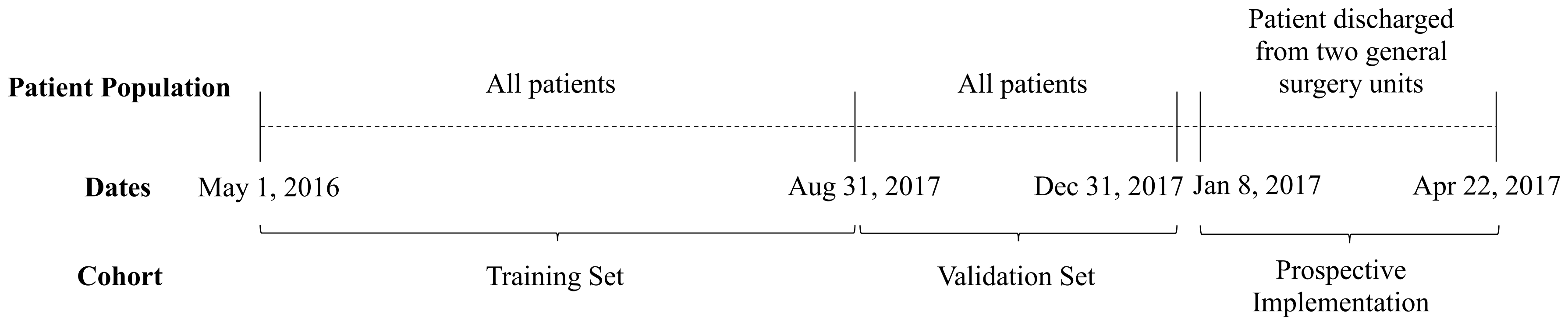

© 2019 Safavi KC et al. *JAMA Network Open.*
